# Profit and loss analysis for an intensive care unit (ICU) in Japan: a tool for strategic management

**DOI:** 10.1186/1472-6963-6-1

**Published:** 2006-01-11

**Authors:** Pengyu Cao, Shin-ichi Toyabe, Toshikazu Abe, Kouhei Akazawa

**Affiliations:** 1 Division of Information Science and Biostatistics, Department of Medical Informatics and Pharmaceutics, Niigata University Graduate School of Medical and Dental Sciences, Asahimachi-dori 1-754, Niigata 951-8520, Japan; 2 Department of Medical Informatics, Niigata University Medical and Dental Hospital Asahimachi-dori 1-754, Niigata 951-8520, Japan

## Abstract

**Background:**

Accurate cost estimate and a profit and loss analysis are necessary for health care practice. We performed an actual financial analysis for an intensive care unit (ICU) of a university hospital in Japan, and tried to discuss the health care policy and resource allocation decisions that have an impact on critical intensive care.

**Methods:**

The costs were estimated by a department level activity based costing method, and the profit and loss analysis was based on a break-even point analysis. The data used included the monthly number of patients, the revenue, and the direct and indirect costs of the ICU in 2003.

**Results:**

The results of this analysis showed that the total costs of US$ 2,678,052 of the ICU were mainly incurred due to direct costs of 88.8%. On the other hand, the actual annual total patient days in the ICU were 1,549 which resulted in revenues of US$ 2,295,044. However, it was determined that the ICU required at least 1,986 patient days within one fiscal year based on a break-even point analysis. As a result, an annual deficit of US$ 383,008 has occurred in the ICU.

**Conclusion:**

These methods are useful for determining the profits or losses for the ICU practice, and how to evaluate and to improve it. In this study, the results indicate that most ICUs in Japanese hospitals may not be profitable at the present time. As a result, in order to increase the income to make up for this deficit, an increase of 437 patient days in the ICU in one fiscal year is needed, and the number of patients admitted to the ICU should thus be increased without increasing the number of beds or staff members. Increasing the number of patients referred from cooperating hospitals and clinics therefore appears to be the best strategy for achieving these goals.

## Background

Intensive care units (ICUs) of university hospitals and advanced medical centers are indispensable for providing critical and intensive care for patients who have either undergone major surgery or who have received emergency care. Hospitals can obtain greater revenue from national insurance by a short admission in the ICU than by admission in other hospital departments. For example, the average length of ICU stay for postoperative patients who have undergone coronary artery bypass grafting (CABG) is 3.5 ± 2.5 days (average ± SD) in our hospital. The medical revenue for CABG is US$ 3,122, which is expressed in purchasing power parity (PPP) by World Health Organization (WHO) US dollars, and the revenue for postoperative admission in the ICU is US$ 888 per day. The latter is much higher than the revenue of US$ 247 per day in the case of postoperative admission in a department other than the ICU. However, the cost of ICU stay for a patient who has undergone CABG at our hospital is estimated to be US$ 1,539 per day. As a result, the patient stay in ICU after CABG is not profitable, but it remains unclear as to whether ICU in Japan is profitable or not. There have so far been no reports on the profit performance of ICUs in Japan despite an abundance of such reports from other countries [1-9].

The cost estimation and a profit and loss analysis are necessary for health care practice. In this study, we performed a profit and loss analysis of the ICU of a university hospital in Japan based on an estimation of the break-even point (BEP). A BEP analysis is a cost accounting method commonly used to determine how much revenue is necessary to cover the total cost [10]. It has many concrete applications, such as in both a laboratory cost analysis and a telemedicine service cost analysis, and it provides the basis for a profitability analysis of new services in a hospital [11-13]. In this BEP analysis, we used an activity-based costing method (ABC) to allocate an appropriate share of indirect costs to the ICU.

### Definition of terminology

We explain the terminology used in this article as noted below. First, we define "cost" as it is used in this paper. The cost is defined as the amount of money that our hospital must pay to perform medical services in the ICU, for example, medical material expenditures, personnel fees, as well as lighting and heating expenses [1,2]. The cost can be classified into direct and indirect costs. The direct cost is defined as a cost that is directly attributable to the activities [11]. These activities mean all events or transactions that create costs [14-16], and the cost objects mean any item for which a separate measurement of cost is desired [17]. An indirect cost is defined as a cost that is not directly attributable to the activities of a specific cost objective [11,14,17,18]. In the case of the ICU, one such indirect cost includes some of the administrative costs required to run the ICU [3] and the costs of co-medical departments related to the ICU, such as computed tomography (CT), magnetic resonance imaging (MRI) and laboratory rooms.

### Outline of the Japanese health care system

The Japanese health care system, which consists of the Social Health Insurance Organization and the Governmental Health Insurance Organization, covers 100% of the population. Until 2003, a fee-for-service system was used for all medical services in Japan. Seventy percent of all fees were paid by the insurance organizations and the remaining 30% were paid by the patients. The revenue of university hospitals in Japan consists of two main components: reimbursement for medical services and official support from the government [19]. In addition to these revenues, extra fees of about 574 to 790 US dollars per day are paid for an ICU stay.

In 2003, a new reimbursement system was introduced in Japan. In this new system, the reimbursement per day is predetermined for each of the 1860 combinations of diagnoses in ICD10 and the corresponding procedures named the Diagnosis Procedure Combination (DPC) [20]. This Japanese-style prospective payment system (PPS) is currently applied to only inpatients at 133 acute-care hospitals, including 80 university hospitals and 2 national centers. The DPC-specific per-diem payment gradually decreases as the length of stay increases.

## Methods

### Actual conditions of the ICU at our hospital

The data on the size, utilization and patient population of the ICU at our hospital were obtained from the hospital information system. In this study, we calculated the patient days (bed occupancy days) using the "day to day" method. For example, if a patient was admitted at 20:00 on a Monday and discharged at 10:00 on a Wednesday, this would be counted as 2 patient days. If a patient was admitted and discharged on the same day, this would be counted as one patient day [21-23]. The bed occupancy rate was calculated as patient days divided by (beds × 365) [24]. These data were compared with those for both other ICUs in Japan and data regarding other countries, which were obtained from the literature [4-6].

### Revenue calculation

The annual revenue data for the ICU were obtained from the hospital accounting system. The total revenue can be obtained by the summation of the individual medical payments of patients treated in the ICU. The revenues of surgery and surgery-related laboratory tests, medical imaging diagnoses and medications were deducted from the total patient revenue because these revenues belong to the department in which the surgery was performed.

### Cost estimation

The cost estimation for the total cost was divided into the direct cost accounting for the ICU, the indirect cost estimation from the co-medical departments of the ICU, and the hospital overhead costs for the ICU such as the building depreciation cost, lighting, water, cleaning and garbage disposal contracts, and telephone charges.

The direct costs were calculated using the data obtained from the hospital accounting system. The hospital's overhead costs were allocated to the ICU corresponding to the percentage of space, the number of employees and the number of patients.

The ABC method was used for indirect cost accounting. Using the ABC method, all events or transactions that create costs are recognized as "activities" and a specific cost driver, which is an index for allocating indirect costs appropriately to the cost object, corresponds to each activity. In this study, the ICU is the cost object. We identified 14 activities *A*_*i*_, *i *= 1,...,14, which indirectly provided services to ICU as shown in the first column of Table 4. Next, we estimated the total cost *X*_*i *_of *A*_*i *_beforehand, and set the corresponding cost drivers to each activity as shown in the second and third columns of Table 4. The total volume of each cost driver, *D*_*i*_, and the sub-volume of *D*_*i *_for the ICU, *d*_*i*_, were investigated and also showed in Table 4. The indirect cost to be allocated to the ICU from *X*_*i*_, *C*_*i*_, is obtained by the following equation:

*C*_*i *_= *X*_*i *_× *d*_*i *_/ *D*_*i*_.

Finally, the total indirect cost, *C*, allocated to ICU is expressed as:

C=∑i=114Ci.
 MathType@MTEF@5@5@+=feaafiart1ev1aaatCvAUfKttLearuWrP9MDH5MBPbIqV92AaeXatLxBI9gBaebbnrfifHhDYfgasaacH8akY=wiFfYdH8Gipec8Eeeu0xXdbba9frFj0=OqFfea0dXdd9vqai=hGuQ8kuc9pgc9s8qqaq=dirpe0xb9q8qiLsFr0=vr0=vr0dc8meaabaqaciaacaGaaeqabaqabeGadaaakeaacqWGdbWqcqGH9aqpdaaeWbqaaiabdoeadnaaBaaaleaacqWGPbqAaeqaaaqaaiabdMgaPjabg2da9iabigdaXaqaaiabigdaXiabisda0aqdcqGHris5aOGaeiOla4caaa@39B3@

### Profit and loss analysis for the ICU

We performed an annual profit and loss analysis for the ICU with a break-even point analysis [10,25,26]. The results of calculating the revenue and cost using the methods described in sections 3.2 and 3.3 were used. The aim of this analysis was to clarify (1) the structure of the costs in the ICU, (2) the revenue needed to cover total costs, and (3) the necessary operating volume (number of patient days) for the ICU per year.

## Results

### Actual conditions of our hospital and the ICU in our hospital

Niigata University Medical and Dental Hospital is a local central hospital that has 770 beds and serves the 2,470,000 people who live in Niigata Prefecture. The hospital had more than 240,000 inpatient days in 2003, and the average length of hospital stay was 20.5 days. The ICU in the hospital has six beds and 1,549 inpatient days with 417 new inpatients per year. The ICU staff includes six doctors and 17 nurses. In 2003, the average bed occupancy rate was 71% and the average length of ICU stay was 3.8 days. Table 1 and Table 2 show an outline of the ICU in our hospital compared with other ICUs in Japan and ICUs in the USA and UK. Patients with more than 250 types of diseases stayed at the ICU, and 160 of those 250 types of diseases were related to surgery.

### Results of cost accounting

Table 3 shows the total revenues, direct costs and hospital overhead costs for the ICU. The annual revenue of the ICU was US$ 2,295,044. The total costs were US$ 2,678,052 and the total direct costs were US$ 2,379,107, thus accounting for 88.8% of the total costs. The hospital overhead costs were US$ 80,785. Table 4 shows the data used to calculate the indirect costs of the ICU using the ABC method and the results of our calculations. The indirect costs which were calculated using the ABC method are shown in Table 4. The total indirect costs were US$ 218,160.

### Results of the BEP analysis

**Figure 1:**
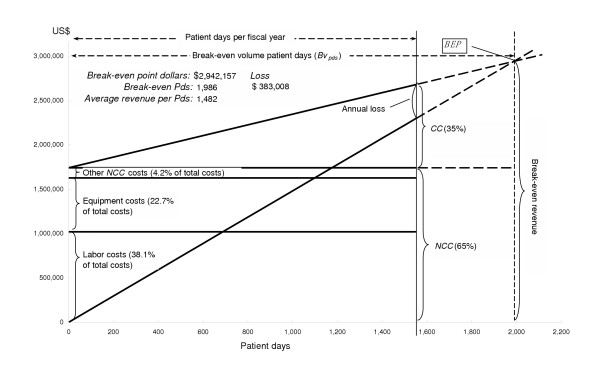
Results of a break-even point analysis for the ICU. BEP: break-even point, *pds *: patient days, *Bv*_*Pds *_: break-even volume patient-days, *CC*: costs that change with the change in the number of patient days, *NCC*: costs that do not change with the number of patient days

The costs that do not vary with the number of patient days were US$ 1,741,380 which was 65% of the total cost. These costs included that 38.1% was labor cost (US$ 1,020,527), 22.7% was equipment cost (US$ 607,189), and 4.2% was the sum of building depreciation, and others expenses costs. The costs that change with the change in the number of patient days were US$ 936,672, which was 35% of the total cost. These costs included that material costs of US$ 735,661 (27.5%), co-medical service cost was US$ 151,871 (5.7%) and other costs (1.8%). The actual number of patient days in the ICU was 1,549. Figure 1 shows the results of a BEP analysis for the ICU. The revenue necessary to cover the costs was US$ 2,942,157. In order to obtain this income, our hospital should increase the number of patient days from 1,549 to 1,986 and increase the average bed occupancy rate from 71% to 91%. These results showed that the ICU will have a deficit operation if the number of patient days can not be increased by more than 437 in the next fiscal year. These findings show that the ICU has to dramatically improve its cost efficiency.

Consequently, the current losses for this fiscal year were US$ 383,008 as estimated from the total revenue and total costs (US$ 2,678,052).

## Discussion

The results of this study based on a profit and loss analysis indicate that ICUs in Japanese hospitals may not be profitable at the present time and that an increase of 437 patient days per year is needed to make up for the deficit in our hospital.

All Japanese national universities, including the attached hospitals, changed over to a new system called the "national university corporation" in April 2004 under the National University Corporation Law. The hospitals are required to carry out self-management and self-responsibility, and will no longer receive national support funding within a few years. Actually, the Japanese government has declared the intention to cut annual national support funding to university hospitals by 2% to 3% and with a goal of a complete cut-off of such funding within five years. Therefore, university hospitals must consider practical strategies to increase the number of inpatients and cut costs.

Reducing the ICU total cost is difficult because of the difficulty in reducing the costs that do not vary with the number of patient days. Almost 88.3% of the total costs were the labor costs of the ICU staff, the equipment costs and the material costs (drugs and medical materials). National university hospitals are obliged to provide the highest and most advanced medical services to patients who stay in the ICU. To maintain and improve the quality of patient care in the ICU, reducing the number of staff members and equipment in the ICU is not a realistic option. Moreover, the cost for inpatients who stay in the ICU of our hospital is almost the same as that in other countries [2,7-9]. Therefore, we should consider strategies to increase the number of ICU inpatients in the setting and circumstances of Japanese national university hospitals.

In 2003, the national university hospitals in Japan had an average bed occupancy rate of 86.4% which thus resulted in many unoccupied beds. To enhance the bed occupancy rate and shorten the length of stay of inpatients, Japanese university hospitals need to strengthen the partnership among university hospital and clinics. In 2003, our hospital had 370,000 outpatients and 240,000 inpatients (patient days), including 1,549 patients in the ICU (patient days). If the total number of patients could increase by more than 10% by increasing the number of patients referred from hospitals and clinics, then an increase of more than 25,000 inpatients (patient days) and more than 170 inpatients (patient days) for the ICU will be possible based on proportional calculations.

Another strategy to increase the number of patients admitted to the ICU is to increase the number of emergency patients. At almost all Japanese university hospitals, the ICU and emergency department are managed as a single unit. With an increase in the number of emergency patients, the number of patients transferred from the emergency room to the ICU would be expected to increase. Emergency inpatients accounted for 20.4% of all inpatients who stayed in the ICU in 2003. Since the emergency outpatients increased another 75% in 2004 in comparison to that in 2003, the unoccupied beds in the ICU could therefore be most easily filled with emergency patients.

We think that the results and implications of this study can be generalized to other ICUs in Japan. Actually, the ICU in our hospital is located in an average position of ICUs in Japan regarding various aspects, as shown in Table 1. The average bed occupancy rate in the ICU of our hospital (71%) is almost the same as that in the ICUs in other hospitals in Japan (72%). In addition to the similarity in the size of the ICU in our hospital and the sizes of ICUs in other hospitals in Japan, there are also similarities in the circumstances surrounding the ICU. A large proportion of ICUs in Japan are managed by university hospitals. Since such hospitals changed to "national university corporations", many of ICUs managed by the national university hospitals will have to face increasing budgetary difficulties. Actions to improve the financial aspects of the ICU are necessary not only for our hospital but also for almost all other university hospitals in Japan.

## Conclusion

The methods of department level ABC for the cost estimation of the ICU and the profit and loss analysis based on BEP analysis are useful to understand that what is the profit or loss for the ICU practice, and how to evaluate and to improve it. In this study, the results indicate that ICUs in Japanese hospitals may not be profitable at the present time. As a result, in order to increase the income to make up for this deficit, an increase of 437 patient days in the ICU in one fiscal year is needed, and the number of patients admitted to the ICU should thus be increased without increasing the number of beds or staff members. Increasing the number of patients referred from cooperating hospitals and clinics therefore appears to be the best strategy for achieving these goals.

## Competing interests

This paper received a grant from the Institute for Health Economics and Policy and the Health Care Science Institute, which provided funding for portions of the study.

No editorial control was allowed.

The article contains nothing that is unlawful, libellous, or which would, if published, constitute a breach of contract or of confidence or of commitment given to secrecy.

## Authors' contributions

Author 1 (PC) designed the study, performed the cost and profit and loss analysis, and drafted the manuscript. Author 2 (ST) conceived of and performed the data collection with the medical data, and participated in drafting the manuscript. Author 3 (TA) performed the data collection related to the management data (e.g., equipments, wages for employees). Author 4 (KA) guided the analysis on hospital management and policy, and participated in designing and drafting the manuscript.

All authors have all read and approved the final manuscript.

## Pre-publication history

The pre-publication history for this paper can be accessed here:



## Figures and Tables

**Table 1:** Organization of intensive care units in Japan, USA and UK

No of ICUs	Our hospital (2003)	Japan (n = 22) (1993–1995)	USA (n = 42) (1993–1995)	UK (n = 17) (1993–1995)
Average number of beds per ICU (% of total beds)	6 (0.8%)	8.0 (1.0 %)	22.0 (4.6%)	4.3
Full-time medical doctors	yes	95.4%	100%	57%
Nurse: patient ratio	1:1	1:1~1:2	1:1	
Average ICU LOS (days)	3.8	6.2	4.7	4.0
Average patient age	50.7	58.3	59.4	60.6
Gender (male: female)	61.4:38.4	64.5:33.5	55.2:44.8	58.4:41.6
Severity of illness (average APACHE III score at day 1)		52.5	50.0	56.8
Post-operative patients (%)	42.4	59.2	42.3	44.1
ICU mortality (%)	6.7		10.0	17.8

Reference		6	6	4,5

**Table 2:** Types of diseases in ICU patients

Disease entity	Number of patients
Blood and blood forming organs	6
Cardiovascular system	185
Digestive systems	53
Infectious diseases	6
Neoplasms	46
Nervous system	9
Poisoning	5
Respiratory diseases	33
Renal diseases	28
Trauma and accidental injury	42
Others	4

Total	417

**Table 3:** Total amount of the revenue of medical payment and official support funding, and those of direct, indirect and the overhead costs for the ICU

Item	Costs (US$)	% of all costs	Total
REVENUE			
Medical payment			2295044
Official support funding			(383008)
COSTS			2678052
Direct cost			(2379107)
Labor cost	1020527	38.11	
Material cost	735661	27.47	
Equipment cost			
depreciation cost	425208	15.88	
repairs cost	45097	1.68	
redemption expenses for equipment investment	136383	5.09	
charge for hire	501	0.02	
Direct expenses			
clothing for employees	788	0.03	
clinical papers, furnishings and other expendables	8011	0.30	
expenses for test contracts	284	0.01	
others	6647	0.25	
Indirect cost (from Table 4)		8.15	(218160)
Hospital overhead			(80785)
building depreciation	18216	0.68	
light, water, heating	18768	0.70	
consignment management	12652	0.47	
others	31149	1.16	

**Table 4:** Data necessary for ICU indirect cost accounting using the activity-based costing method and results of calculation

Activity	Cost of activity	Cost driver	Number of cost drivers	Sub-number of cost drivers for ICU	Indirect costs for ICU (US$)
Supply drugs	1462403	Number of prescriptions	191326	6613	50547
Supply materials	387647	Number of bills	3266	34	4032
Biochemical tests	280277	Number of tests	1712125	18717	3064
Hematological tests	19676	Number of tests	652786	6655	201
Immunochemical tests	11575	Number of tests	223278	2611	135
Other tests	1219546	Number of tests	744919	10057	16465
CT	489443	Number of CT tests	12612	425	16500
MRI	272813	Number of MRI tests	5820	246	11541
Nuclear medical tests	434507	Number of tests	4532	143	13689
X-ray	658632	Number of X-ray tests	104092	2659	16825
Claim remuneration	2857356	Time required	102336	676	18872
Transfer report	2429306	Number of HIS^a^terminals	620	6	23509
Nurse management	243560	Number of nurses	478	17	8662
Hospital management	1491230	Number of employees	1049	24	34118

Total					218160

^a ^HIS: Hospital Information System.
